# Population Genetic Structure and Colonisation History of the Tool-Using New Caledonian Crow

**DOI:** 10.1371/journal.pone.0036608

**Published:** 2012-05-09

**Authors:** Jawad Abdelkrim, Gavin R. Hunt, Russell D. Gray, Neil J. Gemmell

**Affiliations:** 1 Molecular Ecology Laboratory, School of Biological Sciences, University of Canterbury, Christchurch, New Zealand; 2 Department of Anatomy, Centre for Reproduction and Genomics, University of Otago, Dunedin, New Zealand; 3 UMR 7204 Conservation des Espèces, Restauration et Suivi des Populations, Département Ecologie et Gestion de la Biodiversité, Muséum National d’Histoire Naturelle, Paris, France; 4 Department of Psychology, University of Auckland, Auckland, New Zealand; University of Uppsala, Sweden

## Abstract

New Caledonian crows exhibit considerable variation in tool making between populations. Here, we present the first study of the species’ genetic structure over its geographical distribution. We collected feathers from crows on mainland Grande Terre, the inshore island of Toupéti, and the nearby island of Maré where it is believed birds were introduced after European colonisation. We used nine microsatellite markers to establish the genotypes of 136 crows from these islands and classical population genetic tools as well as Approximate Bayesian Computations to explore the distribution of genetic diversity. We found that New Caledonian crows most likely separate into three main distinct clusters: Grande Terre, Toupéti and Maré. Furthermore, Toupéti and Maré crows represent a subset of the genetic diversity observed on Grande Terre, confirming their mainland origin. The genetic data are compatible with a colonisation of Maré taking place after European colonisation around 1900. Importantly, we observed (1) moderate, but significant, genetic differentiation across Grande Terre, and (2) that the degree of differentiation between populations on the mainland increases with geographic distance. These data indicate that despite individual crows’ potential ability to disperse over large distances, most gene flow occurs over short distances. The temporal and spatial patterns described provide a basis for further hypothesis testing and investigation of the geographical variation observed in the tool skills of these crows.

## Introduction

**Figure 1:**
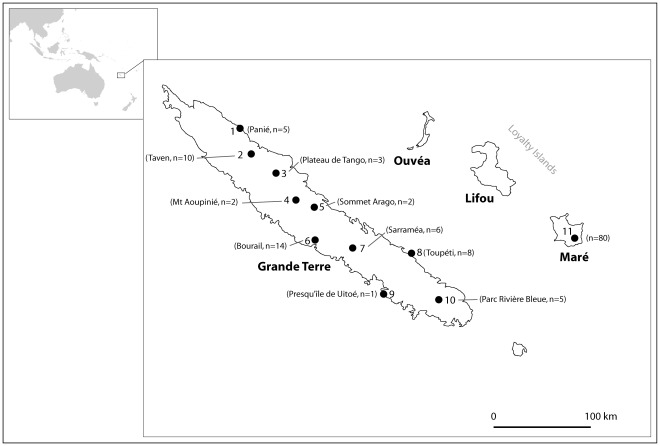
Map of New Caledonia with sample locations. Location of the 11 sites on the islands of Grande Terre, Toupéti and Maré where crow feathers were collected. The samples sizes (*n*) are the numbers of individual crows sampled at the sites.

New Caledonian crows (*Corvus moneduloides*) are omnivorous forest birds endemic to the French Territory of New Caledonia. They live on only two of the larger islands of New Caledonia: Grande Terre and Maré [1] (figure 1). Locals say that crows were introduced to Maré from Grande Terre around 1900 as a biological control for the large endemic katydids (*Pseudophyllanax imperialis*) that were destroying coconut trees (pers. comms. to GRH).

Crows stand out amongst nonhuman animals because of their ability to both manufacture and use tools in seemingly sophisticated ways to extract small prey from vegetation. For example, they are the only species other than humans known to create and use hook tools [2]. In fact, they produce two distinct types of hook tool: those made from live twigs and similar stick-like material [2], [3], and those made from the barbed edges of *Pandanus* spp. leaves [2], [4].

Pandanus tool technology is unique in providing an artefactual record of tool manufacture at any point in time extending back several years [1]. This is because the exact shape of a manufactured tool remains on the edge of a leaf from which it was removed in the form of a ‘counterpart’. Pandanus tool counterparts, or the sections of missing leaf edge, thus allow a quantitative description of the shapes of pandanus tools and the frequency with which they are made at any particular location. Pandanus tool manufacture occurs throughout Grande Terre and Maré, especially in inland forests little disturbed by humans [5]. The analysis of tool counterparts on Grande Terre has revealed interesting variation between sites in the shapes and varieties of pandanus tools made [5]. The shape of a particular variety of pandanus tool is generally highly standardised at sites (e.g. the number of ‘steps’ on stepped tools) [5], but these standard shapes can vary considerably between sites [1], [5]. This shape structure is especially evident for stepped tools on Grande Terre. There are no obvious environmental correlates (e.g. altitude, location) to explain the variation in the stepped design.

A crucial question is what causes the population variation in New Caledonian crows’ pandanus tool manufacture and use? Juvenile crows have an inherited disposition for basic stick tool use [6], but there is no evidence that they have such a disposition to make pandanus tools. Indeed, Hunt *et al.* [7] proposed that the disposition for basic stick tool use combined with learning is crucial for the development of pandanus tool skills in the wild. Work on the island of Maré found that juveniles were raised for an extended period of time (at least up to the next breeding season) in a close family relationship [8]. Juveniles’ tool skills primarily developed in their first year of life in a learning environment strongly scaffolded by their parents [8]. This type of social system minimises the potential for horizontal transmission of tool information via social learning and promotes its vertical transmission. A reliance on vertical transmission should see a close correlation between the spread of tool skills and gene flow. That is, dispersal with successful reproduction (effective dispersal) would be required as opposed to ineffective dispersal combined with only horizontal transmission. Therefore, the spread of tool skills in the New Caledonian crow may be closely correlated with both gene flow and dispersal dynamics.

Genetic, social and ecological aspects may all play a role in bringing about the observed variation in crows’ pandanus tool skills. An important first step in teasing apart the roles of these three components would be describing the genetic structure of the New Caledonian crow. Finding significant genetic structure associated with variation in a particular behaviour does not necessarily mean that it develops without learning. However, it raises the possibility that genetic dissimilarity might play a role in causing the variation [9]. Deciphering the New Caledonian crow’s genetic structure over its geographical range might help us to (1) formulate hypotheses about the cause of pandanus tool variation between locations, and (2) target appropriate areas for future, intensive behavioural and genetic studies. Here, we provide the first description of population genetic structure across the New Caledonian crow’s geographical range and discuss the possible implications of our findings for explaining the population differences in crows’ tool manufacture and use.

## Material and Methods

### Collection of DNA Samples

We extracted DNA from feathers plucked from New Caledonian crows throughout their range (figure 1). Feather samples were collected from 48 crows on mainland Grande Terre (>16,000 km^2^) across nine sites, which were spread over the island (figure 1). Of the 48 crows, 45 were captured in the wild using a ‘whoosh’ net then immediately released after feather samples were taken. Feathers were also taken from two dead crows found freshly shot by hunters (site 5 in figure 1). The remaining crow was a captive bird in Parc Zoo Forestier, Nouméa that was caught as a chick (site 9 in figure 1; see also [7]). We also collected feathers from eight crows captured on the small inshore island of Toupéti (ca. 5 km^2^ in area) on the south east side of Grande Terre (site 8 in figure 1). Toupéti is separated from Grande Terre by a narrow sea channel approximately 80 m wide. On Maré, we collected feathers from 80 crows in the southern part of the island (site 11 in figure 1). Maré is ca. 642 km^2^ in area and is situated 110 km to the east of Grande Terre.

On Grande Terre we sampled crows at sites that varied with respect to habitat and associated tool-making behaviour. For calculations based on local frequencies the sample size at sites had to be at least five (10 alleles) (this was the case at 5 of the 9 Grande Terre sites: sites 1, 2, 6, 7 and 10 in figure 1). The five crows at Panié (site 1) were caught 20 m from the beach, but there was a forest nearby in which *Pandanus* spp. trees grew. We caught 10 crows at Taven (site 2) in inland forest where *Pandanus* spp. trees were absent. The 14 crows at Bourail (site 6) were also caught close to the sea, at the foot of hills where candlenut trees (*Aleurites moluccana*) were common. Crows in candlenut tree areas use tools year round to extract large wood-boring grubs from dead candlenut wood [3], [10]. Site 7 (Sarraméa) was also in a candlenut tree area, but inland in the central mountain chain. The last mainland site (10) was in Parc Rivière Bleue where crows were known to make pandanus tools [5]. All 48 samples from the nine sites on Grande Terre were used in individual-based calculations or calculations making no a priori assumptions on groupings of individuals. Most of the crows that we sampled came from lower altitudes because birds at higher altitudes in forest occur at lower densities and are particularly difficult to catch.

Feather samples were stored in 80% ethanol prior to DNA extraction. DNA was extracted using a Dneasy Blood and Tissue kit (Qiagen Company). To maximise the recovery of DNA, the tubes containing the feathers were centrifuged at 3000 g for 5 mins. Ethanol was then removed and the tubes were inverted on a bench to dry. The pelleted material was then re-suspended in 50 µl of water and added to the buffer/proteinase K mix, along with the feather tips cut into pieces. DNA was finally re-suspended in a 50 µl volume of T.E.

### Genotyping

We used nine microsatellite markers that we had previously optimised to reliably genotype New Caledonian crows [11]. Six of these markers (Ck.1B6G, Ck.2A5A, Ck.5A5G, Ck.4A3G, Ck.5A5F, Ck.4B6D) were developed for the Mariana crow, *C. kubaryi* [12], and the remaining three (CoBr02, CoBr09, CoBr12) for the American crow, *C. brachyrhynchos* [13]. PCRs were performed in 96-well plates using a TETRAD thermal cycler (MJ Research). The cycling profile consisted of a touchdown PCR with one cycle at 94° for 12 mins, 10 cycles of 15 s at 94°, 15 s at 65°, 15 s at 72°, followed by 30 cycles of 15 s at 94°, 15 s at a temperature starting at 65° and decreasing by 0.5° every cycle, and 15 s at 72°. Finally, a 12 min step of elongation was conducted at 72°. Reactions were performed in a volume of 10 µL and contained 2 to 20 ng of genomic DNA, 0.04 µM of Taq polymerase, 1X Buffer, 2.5 µM MgCl2, 0.2 mM dNTP and 0.3 µM of each of forward and reverse primers. The forward primer was modified at the 5′ end by the addition of a fluorescent label (6-Fam, VIC, NED, PET; Applied Biosystems). Labelled PCR products were analysed on an ABI PRISM 3100 Genetic Analyser (Applied Biosystems), and allele sizes were estimated using the Genescan 500 LIZ size standard (Applied Biosystems) in the program GENEMAPPER version 3.7 (Applied Biosystems).

### Statistical Analysis

We used neutral genetic markers to investigate genetic structure in the New Caledonian crow in order to see how genetic variation is structured geographically and to what degree gene flow exists between populations (i.e. dispersal).

The presence of null alleles was tested following Chapuis & Estoup [14] using software FreeNA. To evaluate the level of genotyping error, 10% of the samples were genotyped twice. The power of our set of loci to discriminate individuals was assessed through the estimation of the probability of identity (PI) using Genalex 6 [15]. To have a first grouping hypothesis for our dataset, a clustering analysis was conducted without any *a priori* assumptions on population delimitation. The most likely number of population units (*K)* in the complete dataset (Grande Terre, Maré and Toupéti) was inferred using a fully Bayesian clustering method as implemented in STRUCTURE 2.3.2 [16]. The program was run three times for each value of *K* varying from 1 to 6. We used a classical model with admixture without *a priori* information on population membership with correlated frequencies. Although these models make it easier to distinguish closely related populations, there is a risk of overestimating *K* [17]. After preliminary tests of the convergence time needed for the Monte-Carlo Markov chain, we chose a burn-in period of 100,000 steps followed by 900,000 steps. The most likely value for *K* was estimated using Evanno’s ΔK method [18] using STRUCTURE HARVESTER [19].

Based on the results of this grouping method, standard genetic parameters such as allele frequencies, mean number of alleles per locus and heterozygosity were calculated to estimate genetic diversity. These parameters were computed using the software GENALEX 6 [15]. To correct allelic diversity for difference in sample size, we also calculated allelic richness with Fstat 2.9.3.2 [20], which uses resampling methods based on the smallest number of samples. We performed an exact test of Hardy-Weinberg proportions when there were fewer than five alleles per locus. For five or more alleles, we conducted an unbiased estimate of the exact probability with the Markov chain method of Guo and Thompson [21] for each combination of locus and population using GENEPOP software version 3.3 [22]. We used the sequential Bonferroni method to adjust critical significance levels for simultaneous statistical tests [23] with a nominal significance level of 5%.

We estimated the level of genetic variation between sample sites with at least five samples using Jost’s *D*. We used Jost’s *D* rather than the classical Fst because it is more rigorous at taking into account differences in heterozygosity between sites [24]. The level of significance of Jost’s *D* values was tested using bootstrapping methods with resampling (*n* = 1000). These calculations were carried out using the R package DEMEtics [25]. For completeness, we also provide Fst values to readily enable comparison with other studies given the widespread use of this statistic.

To test for isolation by distance on Grande Terre, we used a Mantel test with *D* as a measure of genetic divergence (tests were also conducted with Fst for comparison) and conducted all combinations of normal and log-transformed distances. The significance of the *Z* statistics was estimated through a randomization procedure (1000 randomizations). Moreover, the strength of the correlation (*r* ^2^) was estimated using a Reduced Major Axis regression. These calculations were performed using IBDWS [26].

We also used the Mantel test to see if there was any geographical pattern in the shapes of stepped pandanus tools that might mirror variation in genetic structure (low numbers of sites prevented us testing for geographical patterns in the shapes of wide and narrow pandanus tools). Specifically, we tested if distance between sites where stepped tools were made on Grande Terre was correlated with variation in the mean length of these tools between the sites. We used 18 of the stepped tool collection sites with adequate samples sizes described in Hunt & Gray [5] for the analysis (sites 1–14 & 16–19). We measured the distance between sites on a 1∶500,000 map. The test was carried out with the function mantel.rtest from the R library ade4. The *p*-value was obtained using permutation methods with 1000 replicates.

We also conducted analyses to determine the genetic relationship between the crow populations on Grande Terre, Toupéti and Maré. We compared the number and identity of alleles for each locus between the two islands to see if Grande Terre was a likely source for the crow population on Maré. We then tested different colonisation scenarios involving the three islands with Approximate Bayesian Computation methods (ABC) using software DIYABC v1.0 [27]. Four scenarios were considered. Scenario one postulated Grande Terre as the source of independent colonisations of Toupéti and Maré, with a time constraint for the later corresponding to a putative single introduction event around 1900 (i.e. 30 to 50 generations for the crows). The second scenario was similar to the first one, but the colonisation event from Grande Terre to Maré was not time constrained. The third scenario tested the possibility of multiple introductions from Grande Terre to Maré; to do this we allowed a second colonisation event. Finally, the fourth scenario involved a linear colonisation sequence first from Grande Terre to Toupéti, then from Toupéti to Maré. The details of the scenarios and parameters used for these analyses are in electronic figure S1. For ease and clarity, the different steps of the ABC analysis are presented jointly with the results in the Results section.

## Results

### Population Delimitation and Genetic Diversity

**Figure 2:**
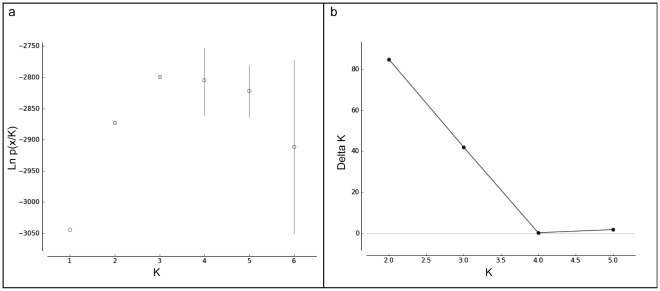
Estimation of the number of cluster identified in New Caledonian crows. (a) likelihood of the number of clusters *K* (mean and standard deviation based on three independent runs) and (b) Variation of ΔK for *K* = 2 to *K* = 5 following Evanno *et al.* [18].

**Figure 3:**
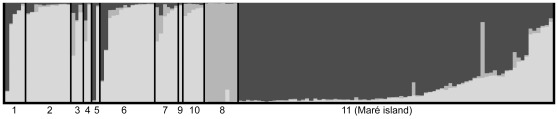
Estimated population structure using clustering methods. Each individual is represented by a vertical line, which is partitioned into segments that represent the individual’s estimated membership fractions in the three clusters. Shades of grey correspond to the three clusters. Black lines separate sampling locations as labelled on the map.

Clustering analysis conducted on the whole dataset indicates that the most likely number of distinct genetic entities is *K* = 3, approximately representing the islands of Grande Terre, Maré and Toupéti (figure 2). The analysis indicated an increased likelihood until *K* = 3, before decreasing (figure 2a). A higher value is obtained for *K* = 2 with Evanno’s ΔK (figure 2b). For *K* = 2, STRUCTURE clearly separates Grande Terre and Toupéti crows from those on Maré (data not shown). For *K* = 3, crows from Toupéti are separated from those on Grande Terre (figure 3). From these results we choose *K* = 3 as the hypothesis for the main population groupings for three reasons: (1) Evanno’s method is known for underestimating *K* [28], (2) individuals from Toupéti are clearly distinct when increasing *K* from 2 to 3, and (3) the mean likelihood gives an optimal value for *K* = 3.

No significant null alleles were detected in the three populations (Grande Terre, Toupéti and Maré). Moreover, the probability of identity dropped under 10^−3^ for as few as five loci. Genotyping errors were low and approximate zero. We found no discrepancies in the 10% of the data that were genotyped twice to test for errors. Global departures from Hardy-Weinberg (table 1) were detected on Grande Terre and Maré, but were due to a restricted number of loci in both cases (i.e. only one and three loci, respectively, showed heterozygote deficiency). The mean number of alleles per locus was higher on Grande Terre than on both Maré and Toupéti (5.78, 5 and 2.44, respectively). This situation was still observed when correcting for the difference in sample sizes, as shown by allelic richness (3.78, 3.55 and 2.4, respectively). When comparing only allelic richness between Grande Terre and Maré, thus aligning sample sizes on Grande Terre (*n* = 40) rather than on Toupéti (*n* = 8), the difference in genetic diversity increases (5.8 and 4.6, respectively). Ten private alleles were found on Grande Terre, three on Maré and none on Toupéti.

**Table 1:** Genetic diversity and heterozygosity in New Caledonian crows on Grande Terre, Toupéti and Maré.

Location	N	Na	Rs	Np	Ho	He
Grande Terre	39	5.78	3.78 (5.8)	10	0.578*	0.621
Toupéti	7.6	2.44	2.4	0	0.437	0.41
Maré	73.4	5	3.55 (4.6)	3	0.566*	0.616

Values of allelic richness in brackets are calculated only with Grande Terre and Maré samples.

N: Mean number of samples per locus; Na: Mean number of alleles per locus; Na: Mean number of alleles per locus; Rs: mean allelic richness per locus; Np: Number of private alleles; Ho: observed heterozygosity; He: expected heterozygosity; *departure from Hardy-Weinberg equilibrium.

### Genetic Differentiation and Isolation by Distance

To quantify the level of genetic differentiation in the New Caledonian crows that we sampled, we computed Jost’s *D* between each pair of the five sites that had at least five samples (table 2). Only two comparisons (Panié vs Taven: *D* = 0.01; Panié vs Bourail: *D* = 0.048) were not significantly different. Excluding Toupéti, values of differentiation on Grande Terre ranged from 0.085 to 0.199. Comparisons between Toupéti and the five Grande Terre sites showed higher values ranging from 0.316 to 0.504. These values are even higher than that obtained for the comparison between Maré and the Grande Terre sites, which ranged from 0.13 to 0.358. The highest value of genetic divergence was between Maré and Toupéti (0.519).

**Table 2:** Genetic differentiation measured by Jost’s *D* (above diagonal) between pairs of locations with at least five samples.

	1.Panié	2.Taven	6.Bourail	7.Sarraméa	8.Toupéti	10.Parc R.B.	11.Maré
1.Panié	–	0.001	0.048	**0.085**	**0.503**	**0.167**	**0.13**
2.Taven	0.008	**–**	**0.198**	**0.118**	**0.503**	**0.197**	**0.218**
6.Bourail	0.025	**0.127**	**–**	**0.101**	**0.504**	**0.199**	**0.229**
7.Sarraméa	0.079	**0.090**	0.057	**–**	**0.316**	**0.091**	**0.174**
8.Toupéti	**0.313**	**0.310**	**0.271**	**0.226**	**–**	**0.345**	**0.519**
10.Parc R.B.	**0.155**	**0.184**	**0.110**	0.077	**0.236**	**–**	**0.358**
11.Maré	**0.081**	**0.113**	**0.113**	**0.082**	**0.260**	**0.176**	**–**

Fst values are indicated below diagonal.

Values in bold are significantly greater than zero (*p* value <0.05).

We also tested for isolation by distance using the five Grande Terre sites with larger sample sizes and Toupéti (table 3). When Toupéti was excluded, isolation by distance is significant for all combinations of distances and log-tranformed distances (*r* ^2^ values ranged from 0.321 to 0.499). When Toupéti is included, isolation by distance is only significant when both distances are log-transformed, resulting in a lower *r* ^2^ (0.246). Similar results are obtained when using Fst as a measure of genetic distance rather than Jost’s D (data not shown). These results indicate two important findings: (1) a correlation between geographical separation and genetic similarity exists on Grande Terre, and (2) the crow population on Toupéti does not fit well into this correlation, possibly because the narrow sea channel between Toupéti and Grande Terre creates a much more restricted gene flow than occurs on Grande Terre.

**Table 3:** Correlation between genetic (Jost’s *D*) and geographic distances on Grande Terre with and without Toupéti.

	with Toupéti		without Toupéti	
analyses	*p*	*r* ^2^	*p*	*r* ^2^
Gendist/GeoDist	0.189	0.044	0.048	0.323
Gendist/Log(GeoDist)	0.111	0.061	0.039	0.375
Log(Gendist)/GeoDist	0.056	0.142	0.031	0.321
Log(Gendist)/Log(GeoDist)	0.02	0.246	0.031	0.499

The strength of the correlation (*r* ^2^) is estimated using reduced major axis regression and the significance (*p*) of the *Z* statistics is evaluated through randomization procedure. All combinations of normal and log-transformed distances are reported.

We found no significant correlation across the 18 stepped-tool making sites between geographical distance and variation in mean tool length (*p* = 0.879, *r* ^2^ = −0.09).

### Colonisation History

To understand the demographic and colonisation history of the three delimited genetic entities (i.e. Grande Terre, Toupéti and Maré), we tested four scenarios using DIYABC (see table 4 and electronic figure S1 for details of the scenarios and their parameters). First, we built a reference table with one million simulated datasets for each scenario, with the default mutation models for microsatellites corresponding to a GSM. All one sample summary statistics were used, along with Fst and the classification index. The adequacy between the scenarios and the priors, as defined in table 4, was evaluated through a principal component analysis (PCA) of the first 100 000 simulated datasets of the reference table in the space of summary statistics. The observed dataset was clearly surrounded by simulated data sets, indicating that our model was able to produce data sets similar to the observed one (data not shown).

**Table 4:** Prior and posterior distributions of demographic and historic parameters used in ABC analyses.

parameters	prior distributions		posterior distributions			
	conditions	distribution	mean	median	quantile 2.5%	quantile 97.5%
						
NGT	–	Uniform [10–20000]	5279	4566	1501	13285
NM0	–	Uniform [10–10000]	4671	4546	193	9709
NT	–	Uniform [10–500]	265	256	66	484
NM1	–	Uniform [5–500]	226	218	54	450
teuro	–	Uniform [30–50]	41	41	31	50
db	–	Uniform [1–100]	52	50	7	98
t1	–	Uniform [1–1000]	–	–	–	–
t2	t2> t1	Uniform [1–1000]	280	232	46	794
M	M < t1	Uniform [1–500]	–	–	–	–
r	–	Uniform [0.001–0.999]	–	–	–	–

Posterior distributions are estimated for the most likely scenario (i.e. scenario 1). The mean and median are given, along with 95% credibility intervals. All times (teuro, t1, t2, db and M) are in generations.

NGT: current effective size of Grande Terre.

NM0: current effective size of Maré.

NT: current effective size of Toupéti.

NM1: effective size of Maré during the post-colonisation bottleneck.

teuro: constrained colonisation time on Maré around 1900 in scenario 1, corresponding to 30 to 50 generations.

db: duration of the post-colonisation bottleneck.

t1: time before present of colonisation of Maré in scenario 2, 3 and 4.

t2: time before present of colonisation of Toupéti.

M: time of second colonisation on Maré in scenario 3 after the first one at t1.

r: admixture rate during the second colonisation event on Maré in scenario 3.

We compared scenarios by computing their posterior probabilities using a logistic regression approach based on the best 40,000 datasets (1%). The logistic regression favoured scenario 1 (i.e. independent colonisations from Grande Terre to Toupéti and Maré, with a constraint on the colonisation time of Maré at around 1900; table 5), closely followed by scenario 2 (*p* = 0.383 and *p* = 0.347, respectively). Scenario 2 provides the same colonisation pattern as scenario one, but with a larger time constraint (0 to 1000 generations instead of 30 to 50). We next estimated the posterior distribution of the parameters used in our model (table 4). We have not undertaken a detailed examination of these posterior distributions, as their 95% credibility intervals were relatively large. Nevertheless, they indicate that the colonisation of Toupéti took place before crows arrived on Maré. Also, the mean of the posterior probability for the colonisation on Maré when the time constraint is relaxed (*t1* in scenario 2, data not shown) still stays close to *teuro* with a mean of 67 generations even though it could potentially have had a value from 1 to 1000. The estimation of NM1 (i.e. the mean number of individuals post-introduction on Maré) is relatively large at more than 200 individuals.

**Table 5:** Relative posterior probabilities with 95% credibility intervals for each scenario using logistic regression approaches.

scenario	logistic regression	
	*P*	95%
1	0.383	[0.3555, 0.4093]
2	0.347	[0.3244, 0.3697]
3	0.261	[0.2397, 0.2839]
4	0.009	[0.0066, 0.0108]

The logistic regression used to compute posterior probabilities considered the 40 000 simulated data sets closest to the observed data (1% of the total number of simulations performed for the four scenarios). Scenario 1 is favoured among the four competing scenarios, closely followed by scenario 2.

We evaluated the goodness-of-fit of scenario 1 by simulating 10,000 datasets under scenario 1 followed by a PCA. This enabled us to verify that the observed data set was well within the range of values obtained through the previous simulations (data not shown). Finally, we evaluated the confidence in scenario choice by estimating type I and type II errors. We first computed 100 datasets under each competing scenario (i.e. a total of 400). Then we calculated (1) the number of times scenario 1 did not have the highest posterior probability when it was the true scenario (Type I error), and (2) the number of times scenario 1 had the highest posterior probability when the true scenario was either scenarios 2, 3 or 4 (type II error). The results suggested that our methodology to discriminate between the four competing scenarios was rigorous (type I error rate was 0.08 and the mean type II error rate was 0.032).

## Discussion

Using nine neutral markers, we conducted the first study of the population genetic structure of the tool-using New Caledonian crow over its geographical range. Our analyses produced four main findings: (1) significant genetic differentiation between sites on Grande Terre, (2) a general signal of isolation by distance on Grande Terre, (3) populations on the islands of Grande Terre, Toupéti and Maré are distinct genetic entities, and (4) confirmation that both Toupéti and Maré populations were founded from Grande Terre.

Our finding that crows on Maré are significantly genetically differentiated from those on Grande Terre was not unexpected given the 110 km of open water between the two islands that should have prevented the birds mixing. The genetic composition of the Maré population is almost a strict sub-sample of the genetic diversity found on Grande Terre, however. Our colonisation scenario analysis is also in agreement with the anecdotal evidence that birds were introduced to Maré from the mainland after European colonization began ca. 1850 [29]. Nevertheless, the level of genetic diversity on Maré is still relatively high, with our scenario testing suggesting that a mean of over 200 individuals was necessary to explain the current genetic diversity of Maré crows. This number of founders seems quite unlikely and should be seen as an indication rather than an absolute value considering the large confidence interval around the mean.

The genetic distinctness of the small crow population on the small inshore island of Toupéti is also a robust finding. Despite crows’ ability to fly, the genetic data suggests that the birds on Toupéti rarely mix with those on Grande Terre. New Caledonian crows are tropical forest birds and as such may be extremely reluctant to cross even a narrow expanse of open water [30]. That such a small population of crows very close to Grande Terre can apparently remain so isolated is surprising, especially if the current population arrived before crows were introduced to Maré around 100 years ago, as our colonisation analyses suggests.

A recent study by Rutz *et al.* [31] on crows from the central west coast of Grande Terre has also shown a significant level of genetic differentiation for locations only a few kilometres apart. While the Fst values they observe are low (but significant) and compatible with the larger values we observe at a larger scale, it is surprising that given their findings we do not detect clear boundaries between populations at a larger scale using similar clustering methods. The different findings of the two studies are likely explained by two key factors. First, an over-representation of related individuals in the Rutz *et al.* [31] study may have resulted in an over-estimation of the number of clusters (intensive sampling was conducted in limited areas). Second, the relatively small sample sizes for each location in our study might have decreased the power of the clustering algorithm resulting in us only being able to detect the most differentiated groups of individuals (i.e. Grande Terre; Toupéti and Maré).

Nonetheless, both studies suggest that genetic structure may exist at fine-scales in New Caledonian crows. Rutz et al. suggest that the genetic structure they found demonstrates the potential for genetic and/or cultural isolation to cause variation in tool skills between crow populations. However, caution is needed in extrapolating the genetic structure between their three sites to the Grande Terre as a whole. This is because only one of their three sites was in forest habitat; the other two sites (farmland and holiday settlement) were highly human modified habitat. Dispersal dynamics of crows between human modified and forest habitat is likely to be different from the dynamics within forest. Most of our sampling sites with five or more crows were in forest habitat. Small sample sizes, though, means that we cannot conclude that fine-scale genetic structure is absent in forest crows on Grande Terre. Importantly, in spite of small sample sizes we found weak isolation by distance in forest where the majority of tool manufacture by crows occurs.

The isolation by distance effect that we found enables us to make inferences about the social system of tool making crows in forest habitat on Grande Terre. It suggests that the species’ social system and dispersal behaviour even more than environmental boundaries (e.g. mountains, deforested areas) are responsible for patterns of gene flow. A field study on Maré [32] found that mated pairs lived year round on permanent foraging ranges that often overlapped with those of other mated pairs. Juveniles developed their tool skills in their first year in a learning environment highly scaffolded by parents [8] and second-year juveniles sometimes delayed dispersal from their natal area [32], [33]. There is no evidence that New Caledonian crows are cooperative breeders though [32], which would explain why some older juveniles delay dispersal. The correlation of geographic distance and genetic differentiation on Grande Terre indicates: (1) restricted effective dispersal between crow populations, and (2) effective dispersal is more frequently over relatively short distances than over large distances. This suggests that crows on the mainland probably have a very similar kind of social system to those on Maré that came from Grande Terre [32]. That is, dispersal mostly occurs only locally when juveniles find partners and establish home ranges generally close to their natal areas. Such a social system would facilitate population level specialization of tool skills by both genetic and cultural effects.

The patterns of gene flow that we found on Grande Terre do not allow us to make inferences about the reasons for the geographical variation in crows’ tool skills. However, the gene flow and the likely closely associated dispersal dynamics of crows on Grande Terre raise the possibility of a genetic effect on the observed local specialization of tool skills, along with both ecological and social effects [5]. The finding of a significant correlation between geographical distance and genetic differentiation is especially interesting. If learning has a limited role in what kind of tool skills develop in a young crow, then we would also predict a correlation between geographical distance and dissimilarity of tool skills. Intriguingly, this does not appear to be the case in crows’ pandanus tool manufacture, where raw material has little effect on final tool shape [5]. We found no geographical distance effect on the mean length of stepped tools at sites to mirror the isolation by distance effect in the genetic structure of crows that we sampled on Grande Terre. Langergraber and Vigilent [9] stress that “…it is only when patterns of genetic and behavioural dissimilarity are discordant that we can make inferences about the processes (genetic or cultural) responsible for between-group variation in behaviour”. The genetic and behavioural data that we analysed here were unrelated. Nevertheless, the lack of a geographical pattern in the shapes of stepped pandanus tools enables us to suggest that the genetic structure of pandanus tool making crows may not be closely associated with the geographical variation in the shapes of pandanus tools. This further raises the possibility that learning may play an important role in bringing about the geographical variation in the shapes of these tools [5].

Our work emphasizes the need for detailed behavioural, ecological *and* genetic data to untangle the processes and mechanisms responsible for the variation in crows’ tool skills. Recent work on chimpanzees clearly shows that such data needs to investigate patterns of genetic and behavioural dissimilarity associated with individual behaviours [9], [34], [35]. Increasing sample sizes at existing sites and adding new sites will be an important step in identifying promising locations for these intensive studies in the future. For example, a more comprehensive data set might reveal ‘mainland islands’ of genetic distinctness similar to Toupéti on Grande Terre. Detailed genetic and behavioural studies might then target populations in close proximity with obviously different tool skills where the variation cannot be easily explained by ecological factors. Looking at selected genes potentially linked to particular behaviours rather than using neutral markers would also enable researchers to better investigate relationships between genetics and behaviour. For example, it has been shown that polymorphism in ‘personality genes’ such as the dopamine receptor D4 (DRD4) appears to be associated with inter-individual differences in exploratory behaviours in a variety of species ranging from humans to the great tit (*Parus major*) [36]. Such studies will require detailed behavioural data on individual crows combined with genetic analyses.

## Supporting Information

Figure S1 **Four different colonisation scenarios of New Caledonian islands by crows.** NGT: current effective size of Grande Terre; NM0: current effective size of Maré; NT: current effective size of Toupéti; NM1: effective size of Maré during the post-colonisation bottleneck; teuro: constrained colonisation time on Maré around 1900 in scenario 1, corresponding to 30 to 50 generations; db: duration of the post-colonisation bottleneck; t1: time before present of colonisation of Maré in scenario 2, 3 and 4; t2: time before present of colonisation of Toupéti; M: time of second colonisation on Maré in scenario 3 after the first one at t1; r: admixture rate during the second colonisation event on Maré in scenario 3.
(TIF)
