# Supplementary figures and images for: Population Genetic Structure and Colonisation History of the Tool-Using New Caledonian Crow
Source: PLoS One. 2012 May 9;7(5):e36608. doi: 10.1371/journal.pone.0036608 (PMC3348878; doi:10.1371/journal.pone.0036608)

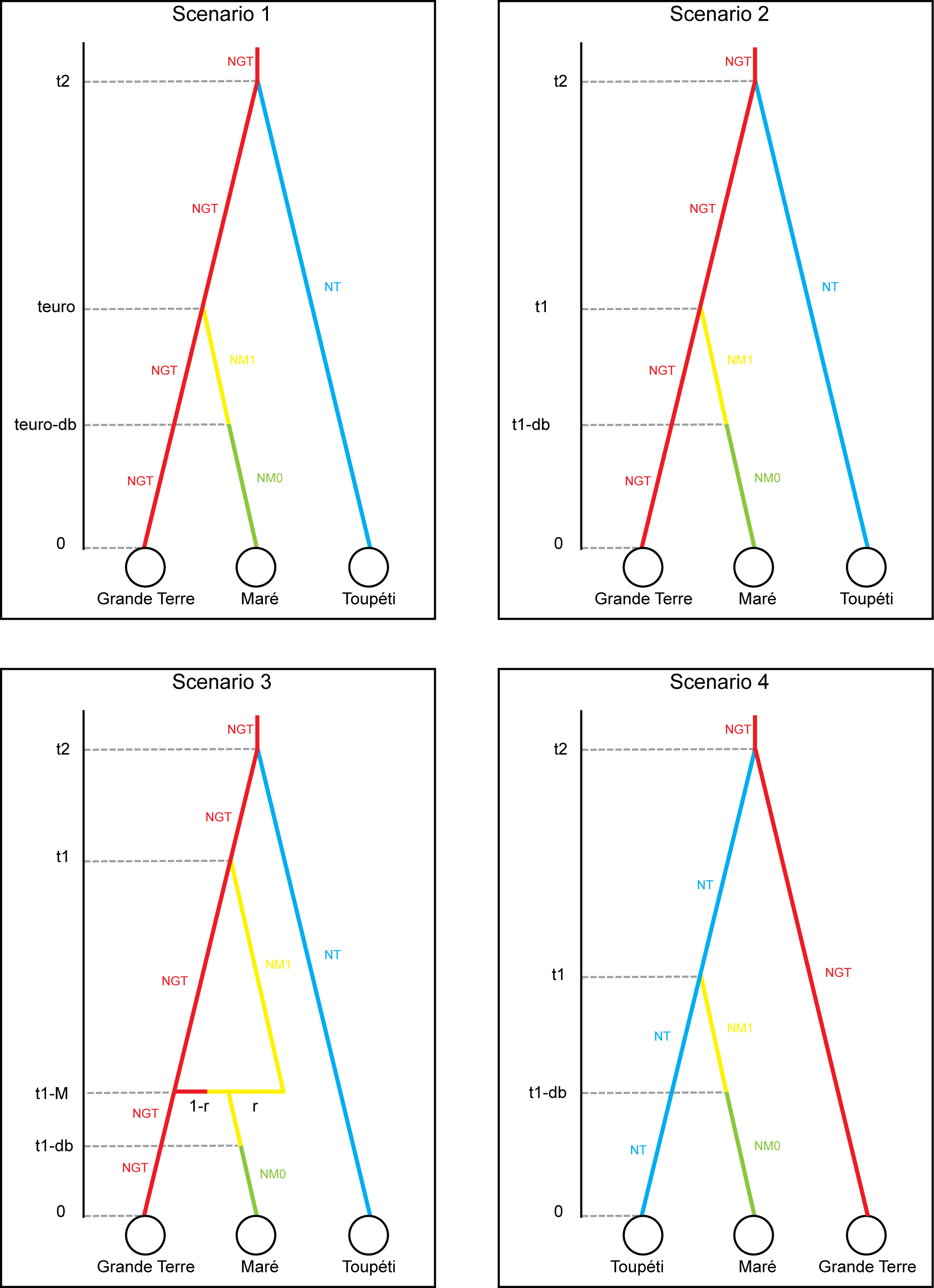

Supplement: Figure S1 — Four different colonisation scenarios of New Caledonian islands by crows. NGT: current effective size of Grande Terre; NM0: current effective size of Maré; NT: current effective size of Toupéti; NM1: effective size of Maré during the post-colonisation bottleneck; teuro: constrained colonisation time on Maré around 1900 in scenario 1, corresponding to 30 to 50 generations; db: duration of the post-colonisation bottleneck; t1: time before present of colonisation of Maré in scenario 2, 3 and 4; t2: time before present of colonisation of Toupéti; M: time of second colonisation on Maré in scenario 3 after the first one at t1; r: admixture rate during the second colonisation event on Maré in scenario 3. (TIF) [file pone.0036608.s001.tif]
